# Measuring loot box consumption and negative consequences: Psychometric investigation of a Swedish version of the Risky Loot Box Index

**DOI:** 10.1016/j.abrep.2022.100453

**Published:** 2022-09-03

**Authors:** David Forsström, Gabriel Chahin, Samuel Savander, Rune A. Mentzoni, Sally Gainsbury

**Affiliations:** a Centre for Psychiatry Research, Department of Clinical Neuroscience, Karolinska Institutet & Stockholm Health Care Services, Region Stockholm, Stockholm, Sweden; b Department of Psychology, Stockholm University, Stockholm, Sweden; c Department of Psychosocial Science, University of Bergen, Norway; d School of Psychology, Brain and Mind Centre, Faculty of Science, University of Sydney, Australia; e Department of Clinical Neuroscience, Karolinska Institutet, Stockholm, Sweden

**Keywords:** Loot boxes, Instrument, Addictive qualities, Psychometric evaluation, Gambling

## Abstract

Loot boxes are products in videogames that is earned by playing a video game or by buying them. Loot boxes has similar mechanisms as a lottery and there is an ongoing debate if loot boxes are gambling. However, to understand the potential harm of loot boxes valid instruments are needed. An English study psychometrically evaluated an instrument focused on risky loot box use called the Risky Loot Box Index. The study evaluated 12 item scale and based on a factor analysis it was reduced to a five-item scale. The aim of our study is to evaluate a Swedish version of the 12-item instrument from a psychometric perspective. Two samples recruited via an online survey were used. The first sample was recruited from the gambling site Unibet. A mail with an invitation to participate was sent to esports bettors and sport bettors at the gambling site. The second sample was recruited from a Facebook forum focused on e-sports. An invitation was posted on the forum to partake in the study. A total of 195 of respondents (96% men and 4% women) with a mean age of 33.76 (SD = 12.34) answered the Unibet survey and 169 respondents (96% men and 4% women and non-binary) with a mean age of 23.89 (SD = 5.52) answered the Facebook survey. An exploratory factor analysis yielded a two-factor seven item solution. The factors were overconsumption regarding time spent on loot boxes and the other factor was focused on overconsumption of loot boxes in terms of spending money. The confirmatory factor analysis conducted in the Facebook sample validated the result from the exploratory factor analysis. The conclusion of the study is that the Swedish version of the Risky Loot Index has good psychometric properties and can used to measure risky loot box consumption.

## Highlights

• Loot Box use and purchases have potentially addictive qualities.
• An instrument, the Risky Loot Box Index, was psychometrically evaluated in a Swedish populations.
• A revised scale consisting of a two factor seven item scale was the best fit.
• The revised scale measures overconsumption in relation time spent on loot boxes and purchases of loot boxes.
• The instrument can be used to measure the potentially addictive qualities of loot box use.
• This is important since loot box use, and purchases has been linked to problem gambling.

## 1 Introduction

Activities such as gaming and watching e-sports are growing exponentially. New ways of producing revenue for the gaming industry have been established, such as loot boxes that are now a part of the monetization in numerous games. Loot box is a type of feature that provides purchase (for real money) of in-game items such as skins (virtual clothes, cosmetic customization for weapons, etc.) or emotes that can be displayed in-game (e.g., in Apex Legends). Several games even offer loot boxes wherein the contents are not purely cosmetic but provide a competitive advantage, such as more powerful game characters (e.g., FIFA and Raid: Shadow Legends) or equipment upgrades (e.g., Diablo Immortal). Typically, the loot boxes can be earned through playing the game, albeit at a slow pace so that purchasing them for real money is the only realistic option to obtain the rarest and most attractive available items. Moreover, it is unknown which prize would be unlocked upon payment and rare items that one acquires can be sold for more money than what the loot box was purchased for. Thus, there is an ongoing discussion on whether loot boxes should be regarded as a form of gambling. The aspect of gambling is linked with loot boxes since it possible to sell rare items that one acquires for more money than what an individual purchased the loot box for. Thus, one can argue that the purchase of loot boxes is similar to gambling based on the definition by Wykes and Berwick (1964) that gambling is the activity or practice of playing a game of chance for money or other stakes. However, not all games offer the possibility of selling the virtual products, making it hard to define the use of loot boxes as gambling. For example, authorities in Belgium have labeled loot boxes as gambling, while the Netherlands do not view it as form of gambling. Nevertheless, the use and purchases of loot boxes can be perilous and are linked with other types of addictive behaviors.

Loot box purchasing is linked with problematic videogaming among adolescents and adults, indicating that it can contribute to other types of problematic behavior (Ide et al., 2021, Zendle et al., 2019). The gambling-like aspects of loot boxes and the observed risks for videogamers demand further investigation into the relation between loot box purchases, gaming, and gambling. One study found an increase in arousal when opening a loot box, suggesting an effect similar to a near miss in gambling (Brady & Prentice, 2021). This supports the addictive potential and gambling-like feature of loot boxes. Spicer et al., 2022, Spicer et al., 2022 found support for a gateway hypothesis regarding loot box use and gambling, wherein loot box purchases lead to gambling and vice versa. A longitudinal study found a positive relationship between problematic videogaming and future problem gambling but did not find the reverse relationship (Molde et al., 2019). A review and *meta*-synthesis found a small correlation between loot box purchases and problem gambling (Spicer et al., 2022, Spicer et al., 2022). However, none of the studies measured problematic loot box use/purchase and other problematic actions to acquire loot boxes but instead used only loot box purchases, which is difficult to link to negative consequences. Therefore, instruments that focus on this lack are required.

Presently, two main instruments measure aspects of loot box purchases. The Reasons And Facilitators For Loot box Engagement scale (Lloyd et al., 2021) measures the motivations for loot box use, whereas the Risky Loot Box Index (RLI) (Brooks & Clark, 2019) investigates risky loot box use and problematic behaviors and actions to acquire loot boxes. The RLI, originally devised as 12-item scale and psychometrically evaluated, is a one-factor solution with five items after several iterations (Brooks & Clark, 2019). However, the instrument needs to be psychometrically investigated for use in different languages. Moreover, there is no clear definition of the construct risky loot box purchase, which warrants further psychometric testing in different samples. Therefore, an instrument with good psychometric properties is required to further examine the link between loot use/purchases and problem gambling. In this context, this study aims to evaluate the RLI from a psychometric perspective by examining factor structure, validity, and reliability for scale. It also explores the correlation with problem gambling.

## 2 Methods

### 2.1 Procedure

The data for this study were collected via two online surveys, both created in LimeSurvey. The first survey was sent out via email in two rounds to customers of the gambling site Unibet and Unibet aided with sending out the email. The content of the e-mail was written by the first author. The first round and second round were sent to individuals who had placed bets on e-sports and engaged in sports betting, respectively. The email contained an invitation to partake in the study and a link to the survey. The survey took approximately 20 min to complete and included approximately 100 questions. It included demographic questions as well as the questions from the Swedish longitudinal gambling study (Romild, Volberg, & Abbott, 2014), questions related to e-sports, the Problem Gambling Severity Index (Ferris & Wynne, 2001), the Patient Health Questionnaire-9 (Kroenke, Spitzer, & Williams, 2001), General Anxiety Disorder-7 scale (Spitzer, Kroenke, Williams, & Löwe, 2006), Alcohol Use Disorders Identification Test (Babor, Higgins-Biddle, Saunders, & Monteiro, 2001), and Drug Use Disorders Identification Test (Berman, Bergman, Palmstierna, & Schlyter, 2005). The Risky Loot Box Index was also administered (Brooks & Clark, 2019).

The second survey was published on an online e-sports forum on Facebook called E-sport Sverige [E-sport Sweden]. The survey included demographic questions (age, gender, and education level) and the Risky Loot Box Index. This survey took approximately three minutes to complete and contained 18 questions. The link for the second survey was posted nine times in the forum from August 2020 to December 2021.

All data was collected anonymously. The respondents agreed to participate without compensation. However, an option was presented to leave their phone number to enter raffle to win a gift card.

### 2.2 Participants

A total of 383 participants responded to the surveys. Nineteen (4.96%) participants, all male, were excluded from the study because they were under the inclusion age of 18 years. They were part of the Facebook respondents.

The final overall sample consisted of 349 males, 14 females and one non-binary with a mean age of 28.54 (SD = 11.04). The sample recruited from Unibet and Facebook consisted of 195 and 169 participants, respectively. Table 1 shows the demographic information.

**Table 1:** Demographic Data.

Demographics	Sample 1: Unibet (Gambling Site) n = 195	Sample 2: Facebook (esports Community) n = 169
Age:		
Mean (SD)	33.76 (12.35)	23.89 (5.52)
Range	52 (18–70)	26 (18–44)
Gender (%):		
Male	187 (95.90%)	162 (95.86%)
Female	8 (4.10%)	6 (3.55%)
Non-binary		1 (0.59%)
Education (%):		
Primary School	15 (7.69%)	15 (8.88%)
High School	74 (37.95%)	103 (60.95%)
Stray Courses (University)	35 (17.95%)	32 (18.93%)
University Degree	71 (36.41%)	19 (11.24%)

Note: Nineteen participants were excluded from Sample 1 due to being under the age of 18.

### 2.3 Measures

The measures presented are the two measures used in the survey. For information about the other measures included in the survey, see supplementary material II.

#### 2.3.1 Risky Loot Box Index

The original version of the RLI is a 12-item questionnaire (see supplementary material for the complete scale) aimed to evaluate a person’s risky use of loot boxes on a 5-point Likert Scale ranging from 1 (strongly disagree) to 5 (strongly agree). The score ranges from 12 to 60 with a higher score indicating riskier use. The original English questionnaire by Brooks and Clark (2019) was translated to Swedish and then back to English to ensure that the accuracy of the translation. The internal consistency of the Swedish version used in this study was 0.921 for the Unibet-sample and 0.825 for the Facebook-sample, which can be interpreted as excellent to good according to George and Mallery (2019). For the items in RLI, see the supplementary material.

In this context, risky use indicates that the use or purchases of loot boxes and the actions to acquire loot boxes have negative consequences for the individual. Several items included in the RLI are similar to those in the Problem Gambling Severity Index (PGSI) (Ferris & Wynne, 2001), which is used to measure problem gambling.

#### 2.3.2 Problem Gambling Severity Index

To assess problem gambling during the previous 12 months, we used the Problem Gambling Severity Index (Ferris & Wynne, 2001). The instrument consists of nine items and the score ranges from 0 to 27. The items are scored from 0 (never) to 3 (almost always). The internal consistency (Omega) for the Unibet-sample was 0.916 showing excellent internal consistency according to George and Mallery (2019). An example item from the scale is: “Have you bet more than you could really afford to lose?”

### 2.4 Statistical analysis

With regard to the number of participants required for an exploratory factor analysis, we followed the *a priori* recommendations by Costello and Osborne (2005) which posit a minimum subject to item ratio of 10:1. Both our samples fulfilled this criterion.

Jamovi V.2.3 and IBM SPSS Statistics v.28 was used to summarize the demographic data and conduct the exploratory factor analysis (EFA) and the correlations. The EFA was based on the Unibet sample, using principal axis factoring (PAF) since the data was skewed. All 12 items were significant according to the Kolmogorov-Smirnov test. The items were skewed toward both low and high numbers on the Likert scale depending on the item. Direct Oblimin rotation was used based on the assumption that the factors yielded were correlated. The retention of factors was based on the outcome of a parallel analysis. Factors were retained if the eigenvalue from EFA was higher than that from the random generated data. Item elimination was performed based on recommendations by Costello and Osborne (2005) regarding communalities (>0.4) and factor loadings (significant at 0.32) as well as on Field (2013) regarding correlations (too high at 0.8).

Pearson correlation analyses were conducted using the Unibet sample to determine if the total score based on the final factor solution was correlated with the total score of the PGSI, the specific item regarding perceived problems following loot box use and age.

AMOS was used for confirmatory factor analysis (CFA) using asymptotically distribution-free method since the data was skewed. The seven remaining items were significant according to the Kolmogorov-Smirnov test. The items were skewed toward both low and high numbers on the Likert scale depending on the item. No errors were correlated between items in the model. The method chosen was based on the recommendations in Huang and Bentler (2015). The CFA was based on the sample from the Facebook online gaming forum. The analysis was made to evaluate whether our proposed factor model from the EFA would hold up in a different sample. The recommendations by Cabrera-Nguyen (2010) on how to report and evaluate the results yielded by the CFA were used. Hu and Bentler (1998) recommendations regarding the interpretation of fit indices were followed while evaluating the CFA results.

Omega was used for internal consistency and average variance and composite reliability was calculated for the Unibet sample.

## 3 Results

### 3.1 Exploratory factor analysis

The initial EFA using Principal axis factoring model yield the following: Kaiser-Meyer-Olkin (KMO) = 0.921; Bartlett’s Test of sphericity, X^2^ = 1988, df = 66, p <0.001, with an internal consistency of α = 0.931. In accordance with Field (2013), we removed the item “My Loot Box use has caused me problems” due to high collinearity (above 0.8). Another four items were excluded: one due to communality below 0.4 and three due to cross loading on both factors (items were double barreled or asked the participants to compare previous and current behavior without benchmarks).

**Fig. 1:**
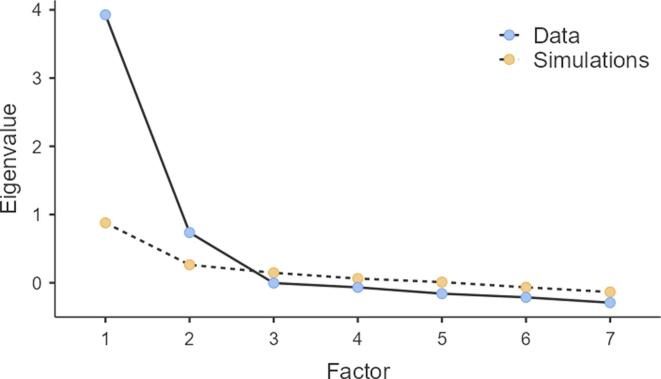
Result from parallel analysis.

The remaining seven items were used in a second EFA, which yielded a two-factor solution (KMO = 0.85; Bartlett’s Test of sphericity, X^2^ = 923, df = 21, p <0.001) with an internal consistency of α = 0.89 suitable for factor analysis. The parallel analysis yielded a two-factor solution with the retained factors having a higher score than random generated data, while the third random score was higher than the EFA eigenvalue (see Fig. 1). The sums of square loadings were 2.76 for factor one and 2.2 for factor two and explained 39.4% and 31.9% of the total variance, respectively. The eigenvalue for factor one was 3.28 and for factor two 0.74.

The items retained pertaining to factor one were (1) I frequently play games longer than I intend to, so I can earn Loot Boxes; (2) I will play for long periods of time to earn Loot Boxes; (3) Receiving items from Loot Boxes is a primary reason why I play video games; and (4) I have put off other activities, work, or chores to be able to earn or buy more Loot Boxes. This factor focuses on the aspect of time with regard to loot box usage. For factor two, the following items were saved: (5) I buy Loot Boxes with the hope of receiving valuable items to sell; (6) I have bought more Loot Boxes after failing to receive valuable items; and (7) The thrill of opening Loot Boxes has encouraged me to buy more. This factor concerns gambling aspects of loot box consumption. For factor loadings of the final model, see Table 2.

**Table 2:** Factor loadings and Communalities of the Final Two-factor Solution.

Items	Time spent regarding loot box usage	Consumption	Communalities
I frequently play games longer than I intend to, so I can earn Loot Boxes	0.80	−0.03	0.60
I will play for long periods of time to earn Loot Boxes	0.91	−0.05	0.78
Receiving items from Loot Boxes is a primary reason why I play video games	0.85	0.06	0.79
I buy Loot Boxes with the hope of receiving valuable items to sell	0.07	0.81	0.73
I have put off other activities, work, or chores to be able to earn or buy more Loot Boxes	0.74	0.06	0.60
I have bought more Loot Boxes after failing to receive valuable items	−0.03	0.90	0.77
The thrill of opening Loot Boxes has encouraged me to buy more	−0.02	0.86	0.72

### 3.2 Confirmatory factor analysis

#### Factor structure

The evaluation of CFA results is based on the recommendations by Cabrera-Nguyen (2010) (see Table 3 for CFA results). We used the sample of individuals from the Facebook gaming forum to evaluate our factor model obtained during EFA. These results indicated an acceptable to good fit. The chi-square value was χ^2^ (13) = 15.64; p =0.269. The root-mean-square error of approximation (RMSEA) was 0.035, indicating a good fit (<0.06) (Hu & Bentler, 1998). Standardized root-mean-square residual (SRMR) was at 0.0642, indicating good fit (<0.08) (Hu & Bentler, 1998). The comparative fit index (CFI) was 0.987 and the Tucker Lewis Index (TLI) was 0.978, indicating good fit in a maximum likelihood CFA which should be close to or above 0.95 (Hu & Bentler, 1998). A correlation analysis between the two factors shows a significant correlation r(1 6 9) = 0.303, p <0.01.

**Table 3:** Factor Loadings from the Confirmatory Factor Analysis with Fixed Parameters.

Factor	Items	Estimate	SE	Z	p
Time spent in regards to loot boxes	I frequently play games longer than I intend to, so I can earn Loot Boxes	1.000*			
	I will play for long periods of time to earn Loot Boxes	1.161	0.114	10.161	< 0.001
	Receiving items from Loot Boxes is a primary reason why I play video games	0.532	0.076	6.954	< 0.001
	I have put off other activities, work, or chores to be able to earn or buy more Loot Boxes	0.450	0.086	5.268	< 0.001
Consumption	I buy Loot Boxes with the hope of receiving valuable items to sell	1.000*			
	I have bought more Loot Boxes after failing to receive valuable items	1.314	0.209	6.292	< 0.001
	The thrill of opening Loot Boxes has encouraged me to buy more	1.232	0.220	5.604	< 0.001

Note: * = fixed parameter. SE = Standard Error. Significance Level (p) = 0.05.

### 3.3 Reliability for the two-factor solution for both samples

For the Unibet sample, Omega coefficient was 0.90 for the revised RLI, 0.90 for the time factor, and 0.895 for the factor regarding monetary consumption to buy loot boxes, showing good internal consistency. The composite reliability and average variance were 0.895 and 0.680 for the time factor and 0.891 and 0.732 for consumption related to money, respectively.

For the Facebook sample, Omega was 0.78 for the revised Risky Loot Box Index, 0.82 for the time factor, and 0.78 for the factor regarding monetary consumption to buy loot boxes, again showing good internal consistency. The composite reliability and average variance were 0.822 and 0.546 for the time factor and 0.719 and 0.465 for consumption related to money, respectively.

### 3.4 Correlation analysis

A correlation analysis showed that the revised RLI had a significant positive correlation with higher scores on the PGSI (r(1 9 3) = 0.186; p =0.009), which signifies a higher degree of reported gambling problems correlated with higher values on the altered questionnaire. Furthermore, correlation analyses were conducted between the revised RLI, time spent with regard to loot boxes, and loot box consumption scores in relation to age.

A correlation was made between the revised RLI and the translated RLI with the removed first item (My Loot Box use has caused me problems) was significantly positive (r(1 9 3) = 0.787; p = <0.001), indicating a higher reported value on the revised RLI correlated with a higher reported value of problems related to loot boxes. For more details, see Table 4.

**Table 4:** Correlation Matrix.

	SWERLI	Time factor	Money factor	PGSI	Age	Item Loot box problems
Revised Risky Loot Box Index	–	–	–	–	–	–
Time spent	0.869**	–	–	–	–	–
Consumption	0.897**	0.560**	–	–	–	–
PGSI	0.186**	0.120	0.204**	–	–	–
Age	−0.150*	0.004	−0.255**	−0.115	–	–
Item Loot box problems	0.787**	0.852**	0.556**	0.137	−0.005	–

Note: * Significant correlation at 0.05. ** Significant correlation at 0.01.

## 4 Discussion

The EFA did not replicate the one-factor structure in Brooks and Clark (2019). Instead, a two-factor solution with seven items was proposed based on the results of the final EFA. Factor one consisted of items that covered time spent on loot boxes and factor two covered money spent on loot boxes. The items retained covered a construct related to overconsumption in terms of time and money spent on loot boxes. Similarities with the overconsumption items in PGSI were also found.

One reason for the different factor structure in our data can be the use of different samples. The sample used in this study was larger than that used in Brooks and Clark (2019) and consisted of gamblers at a gambling site. Arguably, our sample might be a better fit to investigate loot box use than the sample collected at Mturk in Brooks and Clark (2019).

The CFA results supported the two-factor solution that was a result of the EFA. They were in line with the criteria proposed in Hu and Bentler (1998), indicating that the two-factor solution is valid across different populations. However, further studies are required to analyze if the factor structure is valid in different populations. Individuals with gaming disorder should be investigated as they should score high on the revised instrument.

The reliability of the two-factor solution can be considered good to excellent, depending on the sample. The results indicated an overall coherence according to George and Mallery (2019). The two-factor solution had a high correlation with the item “My Loot Box use has caused me problems.” Its high correlation with the revised RLI could be because the question is unspecific but covers if loot boxes cause any problems. The high correlation suggests that factors cover problematic loot box use.

Furthermore, a positive correlation between the revised RLI and PGSI indicated that risky loot box might be linked to gambling problems. However, the correlation was fairly low. Brooks and Clark (2019) also reported a positive correlation between PGSI and the RLI. Several studies, including Spicer et al., 2022, Spicer et al., 2022, have linked risky loot box use and problem gambling and this link should be further explored. It should also be noted that the time factor was not significantly correlated with the PGSI, indicating that spending money on loot boxes, not spending time to earn loot boxes, is associated with problem gambling.

Another interesting aspect of the results is that age and negative consequences from loot box use are inversely correlated. Loot box purchase and use seemingly decrease with age, which is plausible since videogaming might decrease in older age. Perhaps an instrument that targets risky loot box use is more applicable for a population between the age of 15 to 40 years.

### 4.1 Further development of the instrument

Questions regarding negative affect could be included in the instrument to make it similar to PGSI. Items on negative affect were missing in the initial version of the instrument and should be incorporated as negative affect can be a result of overconsumption and indicative of problematic behavior.

Loot box purchases might be more prevalent in a younger population under the age of 18 owing to the extensive videogaming. Perhaps the instrument should be adapted to children and teenagers by adding questions about use in relation to peers. This could also be used to screen adolescents and employ preventive strategies that target gambling if an individual scores high on the questionnaire.

### 4.2 Practical implications

The scale can be used to explore the overconsumption of loot boxes. One way of administering the scale could be to use the question ‘My loot box use is cause for concern’ and dichotomize the item. If the respondents answer ‘yes,’ the entire scale can be administered.

Administering the scale together with instruments covering gambling could provide valuable information about the problems experienced. Furthermore, if there is an overlap between the problem gambling construct, gaming, and problematic loot box use, the scale can be used to collect more information about the construct. The scale can also be used to examine if the pathway from gaming to gambling is present in a sample since individuals that gamble do not start to develop gaming disorder that involves or encompasses loot box use. However, it might be pertinent to administer the revised RLI to populations that have gaming disorder or engage in excessive gaming to obtain an indication of future gambling problems.

### 4.3 Limitations

Self-report bias is likely to be present in the two samples. The overrepresentation of men also constitutes a limitation. A more even gender distribution might have produced a more accurate result both for the EFA and the CFA. Adding to this, the underrepresentation of women and the age restriction in sample II make investigations regarding measurement invariance not plausible. Furthermore, another limitation is the absence of analyses to explore predictive and incremental validity.

### 4.4 Future research

Further studies should investigate the psychometric properties of both the initial RLI and the revised version in a larger sample. Studies with participants under the age of 18 years is also relevant since children are deeply involved in the gaming culture and consume numerous gaming products. It would also be valuable to conduct a Rasch analysis using the items in the original and revised scale to examine how the instrument functions on the item level. Future research should evaluate the instrument using the measurement invariance model to analyze if there is any bias regarding age and gender.

## 5 Conclusions

The Swedish version of the RLI is a valid and reliable instrument that can be used to assess overconsumption and negative consequences of loot box use and to analyze the relationship between loot box overconsumption and gambling.

## CRediT authorship contribution statement

**David Forsström:** Conceptualization, Methodology, Validation, Formal analysis, Investigation, Data curation, Writing – original draft, Visualization, Project administration, Funding acquisition. **Gabriel Chahin:** Formal analysis, Writing – original draft. **Samuel Savander:** Formal analysis, Writing – original draft. **Rune A. Mentzoni:** Writing – review & editing, Supervision. **Sally Gainsbury:** Writing – review & editing, Supervision.

## Declaration of Competing Interest

The authors declare that they have no known competing financial interests or personal relationships that could have appeared to influence the work reported in this paper. This paper was funded by Svenska Spel's research council and the University of Bergen.
