# Supplementary material for: Measuring loot box consumption and negative consequences: Psychometric investigation of a Swedish version of the Risky Loot Box Index
Source: Addict Behav Rep. 2022 Sep 3;16:100453. doi: 10.1016/j.abrep.2022.100453 (PMC9483729; doi:10.1016/j.abrep.2022.100453)
Supplement: Supplementary data 1 [file mmc1.docx]

**Original version of Risky Loot Box Index**

(1) I frequently play games longer than I intend to, so I can earn Loot Boxes.

(2) I believe obtaining items from Loot Boxes is an effective way to generate money.

(3) I will play for long periods of time to earn Loot Boxes.

(4) Receiving items from Loot Boxes is a primary reason why I play video games.

(5) I buy Loot Boxes with the hope of receiving valuable items to sell.

(6) I have felt guilty about the amount of time or money I have spent on Loot Boxes.

(7) I have put off other activities, work, or chores to be able to earn or buy more Loot Boxes.

(8) Once I open a Loot Box, I often feel compelled to open another.

(9) I have sometimes spent more on Loot Boxes than I could afford.

(10) I have bought more Loot Boxes after failing to receive valuable items.

(11) The thrill of opening Loot Boxes has encouraged me to buy more.

(12) My Loot Box use has caused me problems.

Answers range on a five-point Likert scale from strongly disagree to strongly agree.
