# Supplementary material for: Measuring loot box consumption and negative consequences: Psychometric investigation of a Swedish version of the Risky Loot Box Index
Source: Addict Behav Rep. 2022 Sep 3;16:100453. doi: 10.1016/j.abrep.2022.100453 (PMC9483729; doi:10.1016/j.abrep.2022.100453)
Supplement: Supplementary data 2 [file mmc2.docx]

# Other measures in the survey

## Generalized Anxiety Disorder-7 (GAD-7)

The GAD-7 is a self-report measure that measures anxiety (Spitzer et al., 2006). Its internal consistency is excellent (Cronbach’s α = .92), and it has good test–retest reliability (intraclass correlation = .83; Spitzer et al., 2006). The questionnaire includes seven questions and has a single-factor solution. The score ranges from 0 to 21 points and the items range from 0 (“Not at all”) to 3 (“Nearly every day”; Spitzer et al., 2006). The internal consistency (Omega) for the Unibet-sample was .839. An example of a question in the scale is: “Over the last 2 weeks, how often have you been bothered by any of the following problems? Feeling nervous, anxious, or on edge.”

## Patient Health Questionnaire-9 (PHQ-9)

The PHQ-9 is a nine-item instrument measuring depression. It has excellent internal consistency (Cronbach’s α = .89) and a good test–retest correlation (.84; Kroenke et al., 2001). The instrument has a single-factor solution. The score ranges from 0 to 27 points (Kroenke et al., 2001). Items range from 0 (“Not at all”) to 3 (“Nearly every day”). The internal consistency (Omega) for the Unibet-sample was .880. An example of a question in the scale is: “Over the last 2 weeks, how often have you been bothered by any of the following problems? Little interest or pleasure in doing things.”

## The Alcohol Use Disorders Identification Test (AUDIT)

## The AUDIT is an instrument measuring alcohol consumption (Babor et al., 2001). It contains 10 items that are scored from 0-4 on a five step Likert scale with answers differing. The internal consistency (Omega) for the Unibet-sample was .764. An example of a question in the scale is: “How often do you have a drink containing alcohol?”

## The Drug Use Disorders Identification Test (DUDIT)

The DUDIT is an instrument measuring consumption of drugs (Berman et al., 2005) that contains 11 items. As with the AUDIT (Babor et al., 2001) items are scored from 0-4 on a five step Likert scale with answers differing. The internal consistency (Omega) for the Unibet-sample was .882. An example of a question in the scale is: “How often do you use drugs other than alcohol?”

## References

Babor, T. F., Higgins-Biddle, J. C., Saunders, J. B., & Monteiro, M. G. (2001). Audit. *The Alcohol Use Disorders Identification Test (AUDIT): guidelines for use in primary care*.

Berman, A. H., Bergman, H., Palmstierna, T., & Schlyter, F. (2005). Evaluation of the Drug Use Disorders Identification Test (DUDIT) in criminal justice and detoxification settings and in a Swedish population sample. *European addiction research*, *11*(1), 22-31.

Kroenke, K., Spitzer, R. L., & Williams, J. B. (2001). The PHQ‐9: validity of a brief depression severity measure. *Journal of general internal medicine*, *16*(9), 606-613.

Spitzer, R. L., Kroenke, K., Williams, J. B., & Löwe, B. (2006). A brief measure for assessing generalized anxiety disorder: the GAD-7. *Archives of internal medicine*, *166*(10), 1092-1097.
